# High Spin Magnetic Moments in All-3*d*-Metallic Co-Based Full Heusler Compounds

**DOI:** 10.3390/ma16247543

**Published:** 2023-12-07

**Authors:** Murat Tas, Kemal Özdoğan, Ersoy Şaşıoğlu, Iosif Galanakis

**Affiliations:** 1 Department of Physics, Gebze Technical University, 41400 Kocaeli, Turkey; murat.tas@gtu.edu.tr; 2 Department of Physics, Yildiz Technical University, 34210 İstanbul, Turkey; kozdogan@yildiz.edu.tr; 3 Institute of Physics, Martin Luther University Halle-Wittenberg, 06120 Halle (Saale), Germany; ersoy.sasioglu@physik.uni-halle.de; 4 Department of Materials Science, School of Natural Sciences, University of Patras, 26504 Patra, Greece

**Keywords:** Heusler compounds, ab-initio calculations, first-principles, electronic structure, ferromagnetic materials, Slater–Pauling rule

## Abstract

We conduct ab-initio electronic structure calculations to explore a novel category of magnetic Heusler compounds, comprising solely 3d transition metal atoms and characterized by high spin magnetic moments. Specifically, we focus on Co${}_{2}YZ$ Heusler compounds, where *Y* and *Z* represent transition metal atoms such that the order of the valence is Co > *Y* > *Z*. We show that these compounds exhibit a distinctive region of very low density of minority-spin states at the Fermi level when crystallizing in the $L2_{1}$ lattice structure. The existence of this pseudogap leads most of the studied compounds to a Slater–Pauling-type behavior of their total spin magnetic moment. Co${}_{2}$FeMn is the compound that presents the largest total spin magnetic moment in the unit cell reaching a very large value of 9 $\mu_{B}$. Our findings suggest that these compounds are exceptionally promising materials for applications in the realms of spintronics and magnetoelectronics.

## 1. Introduction

In the early 20th century, the German metallurgist Heusler, while searching for ways to enhance the electrical conductivity of steel, made a significant discovery [1,2]. This discovery was a novel compound known as Cu${}_{2}$MnAl. As the 20th century progressed, advancements in instrumentation revealed that Cu${}_{2}$MnAl has a face-centered cubic (f.c.c.) lattice structure, similar to well-known semiconductors such as Si and GaAs. Interestingly, this particular lattice structure is also adopted by a wide range of intermetallic compounds, each possessing unique properties. These intermetallic compounds came to be known as “Heusler compounds” or “Heusler alloys” [3,4]. Notably, several of these Heusler compounds exhibit ferromagnetic properties with high Curie temperatures. They can be categorized into four distinct families based on the number and valence of the atoms they contain: (a) Semi-Heusler compounds, exemplified by NiMnSb, follow the XYZ chemical formula. Here, *X* and *Y* represent transition metal atoms or lanthanides, while *Z* is a metalloid. The lattice structure of semi-Heusler compounds is denoted as “$C1_{b}$”. (b) Full-Heusler compounds, like Co${}_{2}$MnSi, have the chemical formula $X_{2}YZ$, with *X*, *Y*, and *Z* atoms similar to those in semi-Heuslers. These compounds crystallize in the “$L2_{1}$” lattice structure. (c) Inverse Heuslers are similar to full-Heuslers, but the valence of *X* is smaller than that of *Y*. Their lattice structure is known as “XA” or “Xα”. (d) Ordered equiatomic quaternary Heusler compounds, such as (CoFe)TiSi, are represented by the chemical formula $(XX^{\prime })YZ$. They crystallize in the structure called “LiMgPdSn” [4,5]. It is important to note that in all these Heusler compound families, the metalloid atom *Z* plays a significant role.

In the early 21st century, there has been a resurgence of interest in Heusler compounds, primarily driven by the revelation of half-metallicity as a shared characteristic among various ferromagnetic and ferrimagnetic Heusler compounds [6,7,8,9]. Half-metallic compounds exhibit a typical metallic behavior for the majority spin electrons while displaying semiconducting characteristics for the minority spin electrons [10]. This unique property results in a high degree of spin polarization at the Fermi level, making them particularly appealing for applications in the fields of spintronics and magnetoelectronics. They introduce novel functionalities to electronic devices. While other materials have also been explored for their half-metallic properties, Heusler compounds possess distinct advantages due to their elevated Curie temperatures. Consequently, extensive research has been conducted to investigate their fundamental properties and potential applications [11,12,13,14,15]. Notably, recent studies have proposed that certain magnetic Heusler compounds may exhibit even more unconventional behaviors beyond half-metallicity. These include spin-gapless semiconducting and spin-filtering properties, introducing entirely new functionalities to the realm of materials with promising implications for various applications [16,17,18].

The utilization of first-principles calculations, often referred to as ab-initio calculations, represents a potent approach for comprehending the characteristics of examined materials and foreseeing the creation of novel compounds tailored to specific properties. Recently, a growing body of literature has witnessed the emergence of extensive databases that are based on first-principles calculations, encompassing hundreds of magnetic Heusler compounds [19,20,21,22,23,24,25,26]. These compounds hold great promise in the realm of spintronics and magnetoelectronics. These databases serve as a valuable complement to studies that primarily delve into understanding the fundamental origins of these compounds’ properties, focusing on a relatively limited number of Heusler compounds [6,7,8,9].

Modern growth techniques have enabled the realization of thin film compounds that were initially conceived through theoretical predictions. For instance, (CrV)TiAl, a quaternary Heusler compound, was theoretically predicted in Ref. [27] to be a fully-compensated ferrimagnetic semiconductor. Subsequently, it was successfully synthesized, and its distinctive magnetic properties were indeed verified, as evidenced by research in Ref. [28]. In this context, there is a strong rationale for exploring novel Heusler compounds that might exhibit unique properties. As previously mentioned, when it comes to magnetic Heusler compounds, *Z* typically represents a metalloid. However, there are cases, particularly in Heusler compounds that display martensitic transformations, where *Z* can be entirely replaced by a transition metal atom. These particular compounds are referred to as “all-3*d*-metal Heusler alloys/compounds”, as documented in Refs. [29,30,31,32,33,34,35,36,37,38,39,40,41,42,43,44].

Expanding upon this concept within the domain of magnetic Heusler compounds for spintronics applications, we employed in Ref. [45] first-principles electronic band structure calculations to explore the characteristics of a novel category of magnetic all-3d-meta Heusler compounds, specifically, Fe${}_{2}$Cr*Z* and Co${}_{2}$Cr*Z*, wherein *Z* denotes an early 3*d* transition metal like Sc, Ti, or V. These compounds display a pseudogap and exhibit a substantial degree of spin polarization at the Fermi level [45]. Their total spin magnetic moment in the unit cell deviates slightly from the Slater-Pauling rule of perfect half-metallic systems [46]. Notably, it was found in Ref. [45] that compounds such as Co${}_{2}$CrTi and Co${}_{2}$CrV demonstrate robust magnetic properties, primarily attributable to short-range magnetic exchange interactions, resulting in notably high Curie temperatures well above room temperature. Furthermore, these two compounds were found to maintain their magnetic properties even in the presence of B2 disorder, making them highly promising materials for applications in spintronics and magnetoelectronics [45].

Motivated by the above-mentioned results, in the present study, our focus is directed towards Co${}_{2}YZ$ compounds, where *Y* and *Z* are 3d transition metal element such that their valence follows the order Co > *Y* > *Z*. The rationale behind this focus lies in the remarkable high spin magnetic moments exhibited by these compounds, rendering them well-suited for applications in the fields of spintronics and magnetoelectronics. Our research comprehensively examines various facets of these compounds, including their electronic and magnetic properties.

## 2. Computational Details

Our research is dedicated to investigating the ground-state properties of all-3d-metal Heusler compounds. To accomplish this, we employ the full-potential nonorthogonal local- orbital minimum-basis band structure approach (FPLO) for our first- principles electronic band structure calculations, as outlined in Refs. [47,48]. In these calculations, we apply the generalized gradient approximation (GGA) as the exchange-correlation functional within the Perdew–Burke–Ernzerhof (PBE) parametrization [49]. This choice is well-known for yielding precise outcomes, especially when dealing with half-metallic Heusler compounds, aligning closely with experimental observations [6,7]. To ensure the accuracy of our calculations, the total energy is converged to the 10th decimal point. Furthermore, a dense grid of **k**-points, specifically a 20×20×20 grid, conforming to the Monkhorst-Pack scheme [50], is utilized for the integrals in reciprocal space.

**Figure 1:**
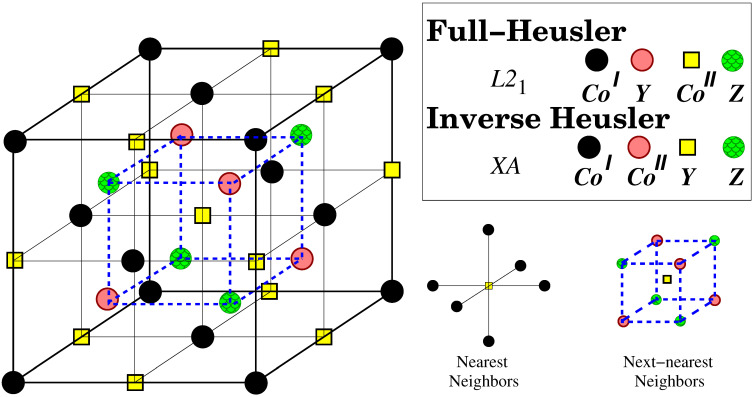
Schematic representation of the $L2_{1}$ structure adopted by the full-Heusler compounds and the XA structure adopted by the inverse Heusler compounds, The black spheres, pink spheres, yellow squares, and green spheres are widely called A, B, C, and D sites, respectively. The large cube in the figure contains exactly four primitive unit cells. On the right, the nearest and next-nearest neighbors are depicted.

Full-Heusler compounds as mentioned above can crystallize either in the regular $L2_{1}$ or inverse XA lattice structures. Both structures are presented in Figure 1. In the regular structure, the two Co atoms sitting at the two different sublattices are equivalent since they have the same environment of nearest neighbors and next- nearest neighbors (the later is rotated by 90${}^{o}$ around the vertical axis). In terms of symmetry the Co atoms in the $L2_{1}$ lattice form a cubic lattice and obey the octahedral symmetry group while the overall symmetry is the tetrahedral one. In the case of the inverse lattice, the situation differs and the two Co atoms are no longer equivalent.

We study all possible compounds having the chemical formula Co${}_{2}YZ$ in both $L2_{1}$ and XA structures using the data from the Open Quantum Materials Database (OQMD) [51]. The lattice constants for the three Co${}_{2}$Cr(V, Ti or Sc) compounds in the regular lattice obtained by OQMD differ less than 1% from the ones calculated using total energy calculations in Ref. [45]. Thus, they can be considered trustworthy. We compile all the results from OQMD in Table 1. Together with the lattice constant in the table we also present the formation energy $E_{\mathrm{form}}$ and the hull distance $E_{\mathrm{hull}}$ in units of eV/atom. For a compound to be stable $E_{\mathrm{form}}$ should be negative meaning that the formation of the compound is favorable with respect to the occurrence of separate bulk crystals made up of a single chemical element. With a few exceptions, notably Co${}_{2}$FeCr, the studied compounds have either negative formation energy or very close to zero. Although the negative value of $E_{\mathrm{form}}$ is a prerequisite, it is not sufficient to decide whether a compound can be grown or not experimentally. $E_{\mathrm{hull}}$ is the energy difference between the assumed lattice structure and the most stable lattice structure or mixture of phases. In all cases, $E_{\mathrm{hull}}$ is positive meaning that none of the two Heusler structures is the most stable phase. Fortunately, for most compounds, the most stable of the two lattice structures ($L2_{1}$ and XA) corresponds to a hull distance of less than 0.200 eV/atom, which is the empirical limit for the experimental growth of a material in a specific lattice structure.

Finally, we should comment on the relative stability of the two possible Heusler lattice structures. In the second column of Table 1 we use a star to denote the most stable lattice structure according to OQMD. For all compounds with the exception of Co${}_{2}$FeCr and Co${}_{2}$MnV, the most stable one is the $L2_{1}$ lattice. We have also performed total energy calculations ourselves and we present our results in the last column of Table 1. Negative (positive) values mean that the $L2_{1}$ (XA) lattice is the most stable. Our results agree with the predictions in OQMD with the sole exception of Co${}_{2}$MnV where our calculations suggest that the $L2_{1}$ and not XA is the most stable lattice structure.

## 3. Results and Discussion

### 3.1. Electronic Properties

**Figure 2:**
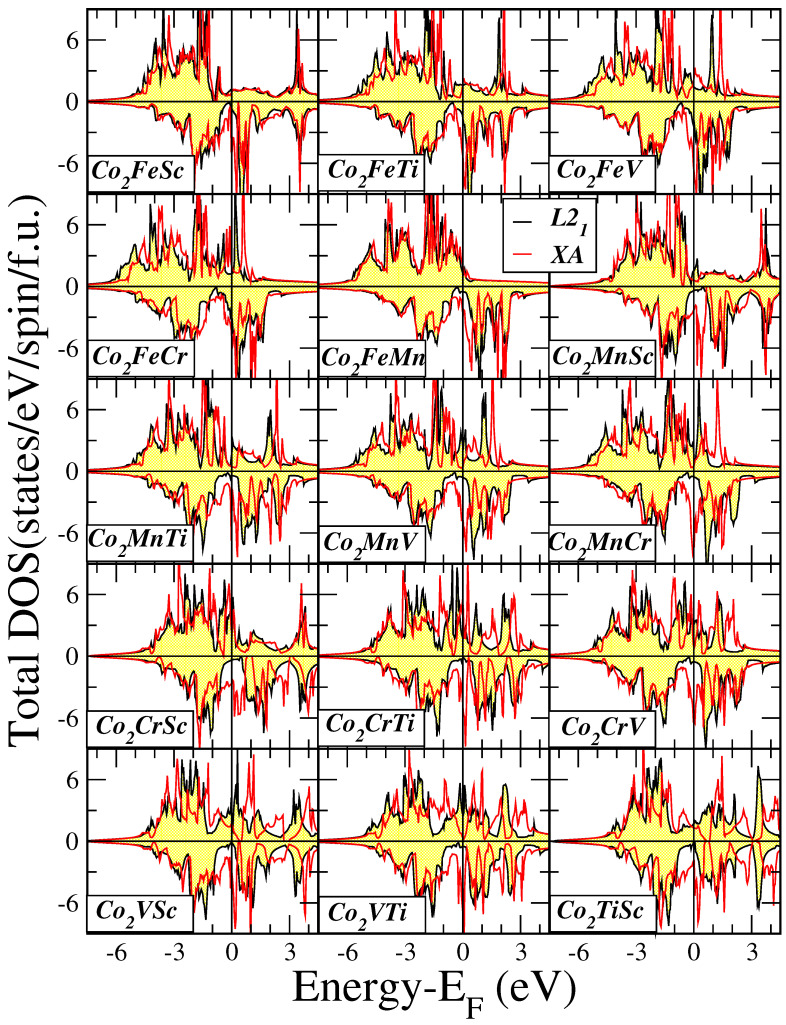
Total density of states (DOS) for all studied compounds for both the $L2_{1}$ and XA lattice structures. Positive (negative) DOS values correspond to the majority (minority)-spin electronic band structure.

At the equilibrium lattice constants, we conducted electronic band structure calculations for all fifteen compounds within our study. Subsequently, we extracted the density of states (DOS) per formula unit (f.u.), which is visually represented in Figure 2. For each compound, we performed a comparative analysis of the total DOS under two distinct lattice structures: $L2_{1}$ (indicated by a yellow background) and XA (illustrated by a red line).

**Figure 3:**
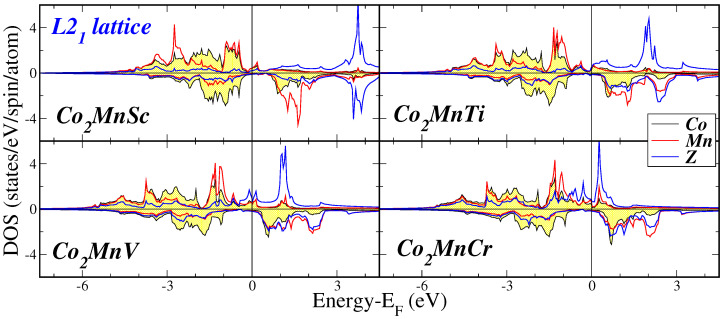
Atom-resolved density of states (DOS) for the Co${}_{2}$MnZ compounds. Details as in Figure 2.

**Figure 4:**
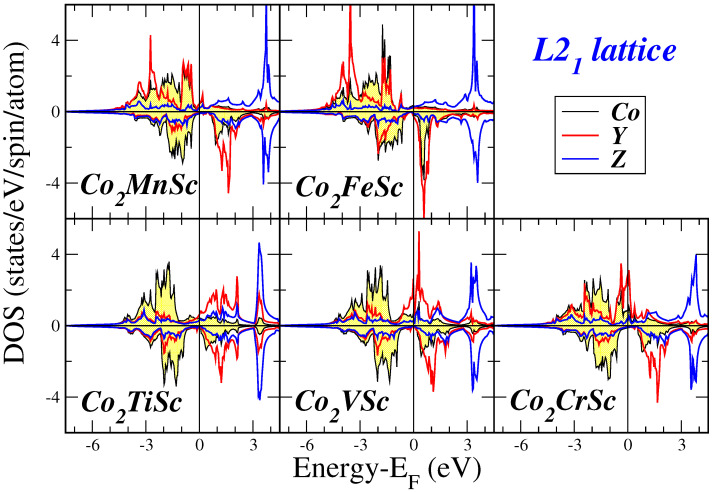
Atom-resolved DOS for the Co${}_{2}Y$Sc compounds. Details as in Figure 2.

When considering the $L2_{1}$ lattice, the DOS exhibits similarities across all compounds examined. Notably, there is a minor DOS intensity at low energy (not displayed in the figures), which primarily originates from the *s*-states. The acquired DOS is predominantly shaped by the 3*d*-states associated with the transition metal atoms, as we will discuss in more detail later. The bands presented in the DOS are notably broad due to the presence of a substantial number of 3*d*-electrons in the f.u. In the majority-spin DOS, the Co atoms possess a substantial number of valence *d*-electrons, resulting in their majority-spin *d*-states being nearly fully occupied as shown in Figure 3 and Figure 4 where we present the atom-resolved DOS for the Co${}_{2}$Mn*Z* and Co${}_{2}$*Y*Sc compounds, respectively. The late transition metal atoms have a considerable number of valence *d*-electrons, causing the majority of the weight of their corresponding majority-spin bands to be situated below the Fermi level. Conversely, early transition metal atoms, including Sc, Ti, and V have a limited number of valence *d*-electrons, causing the majority of the weight of their corresponding bands to be situated above the Fermi level.

**Figure 5:**
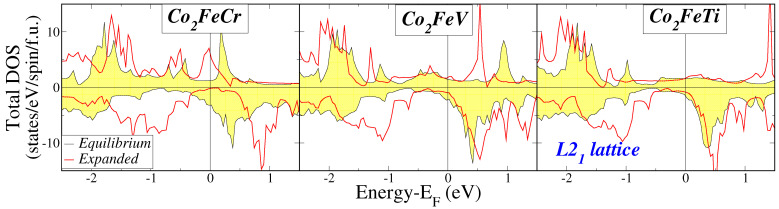
Total DOS for the Co${}_{2}$Fe(Ti, V, Cr) compounds calculated at the equilibrium lattice constant and for a lattice constant expanded by 10%. Details as in Figure 2.

The key observation pertaining to the $L2_{1}$ lattice structures, as depicted in Figure 2 revolves around the energy region proximate to the Fermi level. Across all fifteen compounds, there is a distinct and consistent region characterized by an extremely low density of minority-spin states near the Fermi level, which is commonly recognized as a pseudogap in scientific literature. For most of the compounds, except for Co${}_{2}$Fe(Ti, V, or Cr), the Fermi level falls within this pseudogap. In contrast, for the aforementioned three compounds, the Fermi level positions itself just above the gap. Prior findings concerning half-metallic Heusler compounds have indicated that lattice expansion results in a downward shift of the Fermi level toward lower energy levels, as indicated in Ref. [52]. To shed light on the behavior of the current compounds, we conducted electronic band structure calculations for the three Co${}_{2}$Fe(Ti, V, or Cr) compounds while assuming a lattice constant that is 10% larger than the equilibrium value. The outcomes are presented in Figure 5, depicting the total DOS for both lattice constants. It becomes evident that, for all three compounds, the expansion of the lattice leads to a noticeable shift of the Fermi level towards lower energy values, positioning it squarely within the pseudogap. This shift is particularly pronounced in the case of the Co${}_{2}$FeCr compound.

The total DOS when considering the XA lattice structure differs considerably with respect to the DOS in the $L2_{1}$ lattice structure as evidenced in Figure 2. For all studied compounds there are large peaks around the Fermi level in the minority spin band structure. The Fermi level crosses these peaks resulting in very high DOS values at the Fermi level explaining the reason why the XA structure is unstable with respect to the $L2_{1}$ one with the sole exception of Co${}_{2}$FeCr.

### 3.2. Magnetic Properties

In Table 2, we provide the computed atomic and total spin magnetic moments for all fifteen compounds within our study, employing their equilibrium lattice constants for both considered lattice structures. The Co atoms in all cases carry a spin magnetic moment of around 0.8 to 1.5 $\mu_{B}$ as usually in the compounds containing Co. This is due to the fact that almost all its states are occupied since it has nine valence electrons. In the case of the XA lattice structure, the spin magnetic moments of the two Co atoms vary since their nearest environment (see Figure 1) is different, but deviations are in most cases less than 0.5 $\mu_{B}$. Also, the behavior of the spin magnetic moments of the *Y* and *Z* atoms does not present any peculiarity and follows patterns seen in other magnetic compounds. Interestingly, the early transition metal atoms—Sc, Ti, and V—display spin magnetic moments which are antiparallel to those of the Co atoms. The nature of the coupling between atomic spin magnetic moments varies, depending on the distance and overlap of the 3*d*-wave functions. This coupling can be either ferromagnetic or antiferromagnetic, as dictated by the semi- empirical Bethe–Slater rule [53]. The latter is the case when early and late transition metal atoms are nearest neighbors.

The total spin magnetic moments for both lattice structures are important and in some cases, like Co${}_{2}$FeMn, it even approaches a value of nine $\mu_{B}$ per unit cell which is a very high value. For most of the compounds in the $L2_{1}$ lattice structure, the total spin magnetic moments are close to integer values. This behavior is similar to the behavior of usual half-metallic Heusler compounds crystallizing in the $L2_{1}$ lattice structure [7]. In the next section, we will discuss in detail this behavior investigating its origin.

### 3.3. Slater–Pauling Behavior and Origin of the Pseudogap in the $L2_{1}$ Lattice Structure

**Figure 6:**
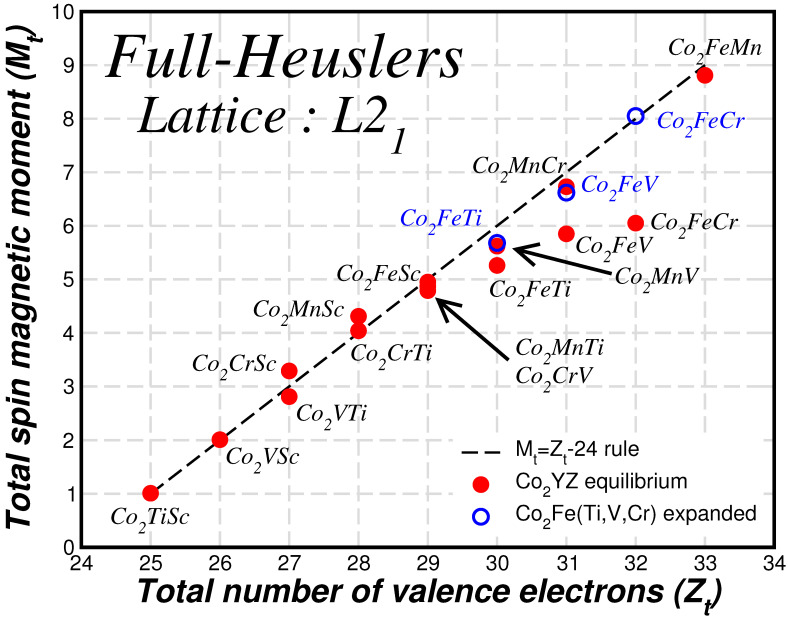
Ab-initio calculated total spin magnetic moments, $M_{t}$, in $\mu_{B}$ as a function of the total number of valence electrons, $Z_{t}$, in the primitive unit cell for the studied compounds at their equilibrium lattice constant assuming the $L2_{1}$ lattice structure (red spheres). With blue empty spheres, we present the results for the three compounds studied at a lattice constant expanded by 10% (see Table 1). The dashed line represents the $M_{t}=Z_{t}-24$ Slater–Pauling rule.

Following the discussion of the total spin magnetic moments per formula unit in Refs. [7,45], we focus now on the behavior of the total spin magnetic moment $M_{t}$ per f.u. in the case of the $L2_{1}$ lattice structure. We have included the corresponding values together with the number of valence electrons $Z_{t}$ in the unit cell, which contains exactly one formula unit, in Table 2. Notably, the values of the total spin magnetic moment for the regular structure are close to integer values. To make it more transparent in Figure 6, we plot the calculated values of the total spin magnetic moments as a function of the total number of valence electrons (red spheres). We remark that with the exception of the three compounds Co${}_{2}$Fe(Ti, V or Cr) for the rest of the compounds the values fall almost on top of the dashed line representing a Slater–Pauling rule of the form $M_{t}=Z_{t}-24$ which resembles the case of the usual regular full-Heusler compounds [46]. This behavior is remarkable since several of the compounds have very large values of spin magnetic moments, e.g., Co${}_{2}$MnCr has a spin magnetic moment of seven $\mu_{B}$ and Co${}_{2}$FeMn a total spin magnetic moment of nearly nine $\mu_{B}$. These values make these compounds of particular interest for applications since they can create very large magnetic fields. Even in the case of the three compounds, which are an exception, if we expand the lattice by 10% the Fermi level falls within the pseudogap (see Figure 5) and the total spin magnetic moment (empty blue sphere) falls almost or exactly on top of the line representing the Slater–Pauling rule. Thus, there should be a unified explanation for this behavior, which associates the pseudogap and the behavior of the total spin magnetic moment when the Fermi level falls within this pseudogap.

**Figure 7:**
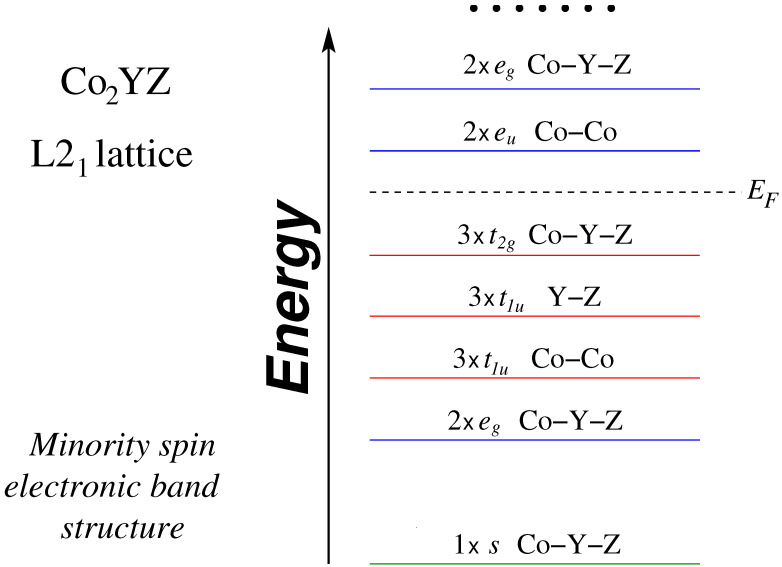
Schematic representation of the character of the bands at the Γ point in the minority-spin band structure of Co${}_{2}$YZ compounds when crystallizing in the $L2_{1}$ lattice structure (see text for details). Note that below the Fermi level, there are 12 orbitals.

To elucidate the origin of the pseudogap we should perform an analysis similar to the one for other half-metallic Heusler compounds [7] and the one in Ref. [45] for the Co${}_{2}$Cr*Z* compounds. The Slater–Pauling rule $M_{t}=Z_{t}-24$ means that there should be exactly 12 fully-occupied minority-spin electronic bands. To characterize these minority-spin bands we performed a fat band analysis for these compounds and in Figure 7 we schematically present the character of the occupied minority-spin bands denoting the band degeneracy at the Γ point, the character of the band, and specifying the atoms whose orbitals contribute to the band. It is important to note that there are two distinct types of 3*d*-orbitals, each corresponding to a specific symmetry, similar to the conventional full Heusler compounds [7]. The overall symmetry for these compounds adheres to the tetrahedral $T_{h}$ symmetry resulting in the “gerade” $e_{g}$ and $t_{2g}$ states which are spread over the whole crystal. Co atoms form a cubic lattice, allowing for “ungerade” states that adhere solely to the octahedral symmetry $O_{h}$. Similarly, the cubic lattice formed by *Y* and *Z* atoms results in a similar arrangement. These “ungerade states” ($e_{u}$ and $t_{1u}$) are localized exclusively either at the Co-Co sites or the Y−Z sites. Finally, we should note that the occupied and unoccupied *d* bands shown in Figure 7 are not separated by an energy gap but overlap away from the Γ point, and thus the Fermi level crosses them resulting in a small deviation as shown in Figure 6 of the total spin magnetic moment from the ideal values predicted by the aforementioned Slater–Pauling rule.

## 4. Summary and Conclusions

By employing ab-initio electronic structure calculations, we introduced an alternative category of magnetic Heusler compounds exclusively composed of Co and 3d transition metal atoms, characterized by high spin magnetic moments. This compound family, Co${}_{2}YZ$, where *Y* and *Z* represent transition metal atoms such that the order of the valence is Co > *Y* > *Z*, exhibit a distinctive region of the very low density of minority-spin states at the Fermi level when crystallizing in the $L2_{1}$ lattice structure. Notably, the total spin magnetic moments per formula unit approximate integer values in most cases and a detailed analysis of the minority-spin band structure unveils that a Slater–Pauling rule can be formulated, and the number of fully occupied bands in the minority-spin band structure is close to 12 in most cases. For compounds deviating from this rule, an expansion of the lattice by 10% shifts the Fermi level within the pseudogap and restores this almost Slater–Pauling behavior. Co${}_{2}$FeMn is the compound that presents the largest total spin magnetic moment in the unit cell reaching an astonishing value of nine $\mu_{B}$.

We expect our results to pave the way for further experimental studies on these compounds, which are susceptible to finding several applications in spintronics and magnetoelectronics.

## Figures and Tables

**Table 1:** The equilibrium lattice constant $a_{equil}$, the formation energy $E_{\mathrm{form}}$ and the hull distance $E_{\mathrm{hull}}$ (data taken from the Open Quantum Materials Database [51]). With star, *, is denoted the calculated ground state in the OQMD. The last column presents the energy difference between the $L2_{1}$ and the XA lattice structures as calculated in the present study.

*XYZ*	Structure	$a_{\mathrm{equil}}$ (Å)	$E_{\mathrm{form}}$ (eV/atom)	$E_{\mathrm{hull}}$ (eV/atom)	$\Delta E^{L2_{1}-\mathrm{XA}}$ (eV/f.u.)
Co${}_{2}$FeSc	$L2_{1}*$	5.884	−0.153	0.123	−0.499
	XA	5.911	−0.033	0.243	
Co${}_{2}$FeTi	$L2_{1}*$	5.727	−0.225	0.039	−0.275
	XA	5.748	−0.167	0.097	
Co${}_{2}$FeV	$L2_{1}*$	5.630	−0.033	0.130	−0.114
	XA	5.644	−0.019	0.145	
Co${}_{2}$FeCr	$L2_{1}$	5.509	0.377	0.414	0.098
	XA*	5.598	0.170	0.207	
Co${}_{2}$FeMn	$L2_{1}*$	5.563	−0.002	0.040	−0.372
	XA	5.616	0.087	0.129	
Co${}_{2}$MnSc	$L2_{1}*$	5.902	−0.169	0.102	−0.735
	XA	5.964	0.005	0.276	
Co${}_{2}$MnTi	$L2_{1}*$	5.759	−0.289	0.010	−0.587
	XA	5.799	−0.154	0.144	
Co${}_{2}$MnV	$L2_{1}$	5.603	0.196	0.365	−0.143
	XA*	5.669	−0.035	0.134	
Co${}_{2}$MnCr	$L2_{1}*$	5.614	0.093	0.122	−0.216
	XA	5.628	0.129	0.158	
Co${}_{2}$CrSc	$L2_{1}*$	5.928	−0.014	0.258	−0.898
	XA	5.971	0.206	0.477	
Co${}_{2}$CrTi	$L2_{1}*$	5.786	−0.152	0.096	−0.703
	XA	5.734	0.014	0.262	
Co${}_{2}$CrV	$L2_{1}*$	5.698	0.029	0.172	−0.236
	XA	5.632	0.072	0.213	
Co${}_{2}$VSc	$L2_{1}*$	5.960	−0.133	0.180	−0.852
	XA	5.944	0.077	0.390	
Co${}_{2}$VTi	$L2_{1}*$	5.806	−0.223	0.085	−0.460
	XA	5.794	−0.125	0.182	
Co${}_{2}$TiSc	$L2_{1}*$	6.041	−0.367	0.028	−1.287
	XA	6.052	−0.004	0.390	

**Table 2:** Atom resolved and total (per formula unit) spin magnetic moments. The last column presents the total number of valence electrons in the primitive unit cell, which contains exactly one formula unit. For Co${}_{2}$Fe(Ti, V, Cr) we have also performed extra calculations for the $L2_{1}$ lattice structure assuming a lattice constant 10% larger than the equilibrium one.

*XYZ*	Structure	$m^{{\mathrm{Co}}^{I}}$ ($\mu_{B}$)	$m^{{\mathrm{Co}}^{\mathrm{II}}}$ ($\mu_{B}$)	$m^{Y}$ ($\mu_{B}$)	$m^{Z}$ ($\mu_{B}$)	$m^{f.u.}$ ($\mu_{B}$)	$Z_{t}$
Co${}_{2}$FeSc	$L2_{1}$	1.24	1.24	2.88	−0.40	4.95	29
	XA	1.19	1.68	2.20	−0.47	4.61	
Co${}_{2}$FeTi	$L2_{1}$	1.33	1.33	2.89	−0.29	5.26	30
	expanded $L2_{1}$	1.61	1.61	3.20	−0.74	5.68	
	XA	1.02	1.61	1.95	−0.63	3.95	
Co${}_{2}$FeV	$L2_{1}$	1.42	1.42	2.79	0.22	5.85	31
	expanded $L2_{1}$	1.63	1.63	3.18	0.18	6.62	
	XA	0.98	1.50	1.90	−0.86	3.52	
Co${}_{2}$FeCr	$L2_{1}$	1.37	1.37	2.50	0.81	6.05	32
	expanded $L2_{1}$	1.33	1.33	3.05	2.34	8.05	
	XA	1.47	1.57	2.23	−0.29	4.99	
Co${}_{2}$FeMn	$L2_{1}$	1.47	1.47	2.83	3.05	8.81	33
	XA	1.60	1.76	2.37	2.75	8.49	
Co${}_{2}$MnSc	$L2_{1}$	0.82	0.82	3.12	−0.44	4.31	28
	XA	1.23	1.56	2.95	−0.42	5.31	
Co${}_{2}$MnTi	$L2_{1}$	1.05	1.05	3.23	−0.45	4.87	29
	XA	1.20	1.61	2.45	−0.65	4.61	
Co${}_{2}$MnV	$L2_{1}$	1.15	1.15	3.07	0.25	5.62	30
	XA	1.12	1.52	2.14	−0.93	3.85	
Co${}_{2}$MnCr	$L2_{1}$	1.23	1.23	2.95	1.32	6.73	31
	XA	0.76	1.53	2.32	−1.71	2.89	
Co${}_{2}$CrSc	$L2_{1}$	0.70	0.70	2.21	−0.032	3.29	27
	XA	1.16	1.29	2.47	−0.24	4.68	
Co${}_{2}$CrTi	$L2_{1}$	0.99	0.99	2.38	−0.32	4.04	28
	XA	0.89	1.13	0.98	−0.17	2.84	
Co${}_{2}$CrV	$L2_{1}$	1.13	1.13	2.25	0.28	4.79	29
	XA	0.76	1.01	0.56	0.09	2.42	
Co${}_{2}$VSc	$L2_{1}$	0.82	0.82	0.60	−0.22	2.01	26
	XA	1.20	1.39	−0.98	−0.19	1.41	
Co${}_{2}$VTi	$L2_{1}$	1.02	1.02	0.83	−0.05	2.81	27
	XA	0.76	0.88	−0.48	−0.22	0.94	
Co${}_{2}$TiSc	$L2_{1}$	0.78	0.78	−0.32	−0.22	1.01	25
	XA	1.32	1.33	−0.51	−0.23	1.90	

## Data Availability

The data presented in this study are available on request from the corresponding author.

## Abbreviations

The following abbreviations are used in this manuscript:

DOS	Density of States
f.u.	formula unit
FPLO	Full-potential nonorthogonal local-orbital minimum- basis band structure approach
GGA	Generalized gradient approximation
PBE	Perdew Burke Ernzerhof
